# Reduced frequency of cytotoxic CD56^dim^ CD16^+^ NK cells leads to impaired antibody-dependent degranulation in EBV-positive classical Hodgkin lymphoma

**DOI:** 10.1007/s00262-021-02956-x

**Published:** 2021-05-15

**Authors:** Elena Pánisová, Anna Lünemann, Simone Bürgler, Monika Kotur, Julien Lazarovici, Alina Danu, Meike Kaulfuss, Juliane Mietz, Obinna Chijioke, Christian Münz, Pierre Busson, Christoph Berger, David Ghez, Tarik Azzi

**Affiliations:** 1 Experimental Infectious Diseases and Cancer Research, University Children’s Hospital of Zurich, Zurich, Switzerland; 2 Children’s Research Center, University Children’s Hospital of Zurich, Zurich, Switzerland; 3 Present Address: Department of Neurology With Institute of Translational Neurology, University of Münster, Münster, Germany; 4 Department of Hematology, Gustave Roussy and Université Paris Saclay, Villejuif, France; 5 Cellular Immunotherapy, Institute of Experimental Immunology, University of Zurich, Zurich, Switzerland; 6 Institute of Pathology and Medical Genetics, University Hospital Basel, Basel, Switzerland; 7 Viral Immunobiology, Institute of Experimental Immunology, University of Zurich, Zurich, Switzerland; 8 CNRS UMR 9018, Gustave Roussy and Université Paris Saclay, Villejuif, France

**Keywords:** Epstein-Barr virus, Classical Hodgkin lymphoma, Natural killer cells, Rituximab, Antibody-dependent cellular cytotoxicity

## Abstract

Around 30–50% of classical Hodgkin lymphoma (cHL) cases in immunocompetent individuals from industrialized countries are associated with the B-lymphotropic Epstein-Barr virus (EBV). Although natural killer (NK) cells exhibit anti-viral and anti-tumoral functions, virtually nothing is known about quantitative and qualitative differences in NK cells in patients with EBV+ cHL vs. EBV- cHL. Here, we prospectively investigated 36 cHL patients without known immune suppression or overt immunodeficiency at diagnosis. All 10 EBV+ cHL patients and 25 out 26 EBV- cHL were seropositive for EBV antibodies, and EBV+ cHL patients presented with higher plasma EBV DNA levels compared to EBV- cHL patients. We show that the CD56^dim^ CD16^+^ NK cell subset was decreased in frequency in EBV+ cHL patients compared to EBV- cHL patients. This quantitative deficiency translates into an impaired CD56^dim^ NK cell mediated degranulation toward rituximab-coated HLA class 1 negative lymphoblastoid cells in EBV+ compared to EBV- cHL patients. We finally observed a trend to a decrease in the rituximab-associated degranulation and ADCC of in vitro expanded NK cells of EBV+ cHL compared to healthy controls. Our findings may impact on the design of adjunctive treatment targeting antibody-dependent cellular cytotoxicity in EBV+ cHL.

**Supplementary Information:**

The online version contains supplementary material available at 10.1007/s00262-021-02956-x.

## Introduction

Classical Hodgkin lymphoma (cHL) is a solid lymphoid cancer characterized by a very low frequency of neoplastic cells, i.e., the Hodgkin and Reed-Sternberg (HRS) cells, that are surrounded by an inflammatory tumor microenvironment (TME) [1]. The pathognomonic HRS cells are of B cell origin with evidence of somatic hypermutation indicating that they are germinal center experienced [2]. cHL can be subclassified into four histologic subtypes (nodular sclerosis, mixed-cellular, lymphocyte-rich and lymphocyte-depleted) and can be staged I to IV depending on tumoral extension. Treatment of cHL with chemotherapy achieves a 5-year cure in more than 80% of the cases [3]. Patients suffering from treatment-refractory cHL might ultimately benefit from novel antibody-based immunotherapies [4].

Around 30–50% of cHL cases in immunocompetent individuals from industrialized countries are associated with the -herpesvirus Epstein-Barr virus (EBV) [5], while in resource-poor countries this association may be higher than 70% and in patients infected with HIV up to 100% [2]. The EBV status of the HRS cell in newly diagnosed cHL is not routinely determined since it does not impact on the choice of chemotherapy regimen in treatment guidelines. EBV establishes an asymptomatic latent infection in the vast majority of adults and is primarily controlled by T cells and natural killer (NK) cells [6]. EBV exhibits potent B cell growth-transformation properties in vitro and is associated with several B cell cancers in immunocompetent and immunocompromised individuals. With respect to cHL, EBV is preferentially linked to the mixed cellularity and lymphocyte-depleted subtypes and is found as monoclonal viral genome in HRS cells [5]. Epidemiological [7] and genetic [8] studies suggest that EBV-negative (EBV-) cHL and EBV-positive (EBV+) cHL differ in their pathogenesis, EBV latent genes thereby providing survival signals for HRS cells.

NK cells contribute to the immune control of malignant cells [9, 10] and viruses [11]. The blood NK cell compartment is mainly composed of the two well-characterized functional CD56^bright^ CD16^-^ and CD56^dim^ CD16^+^ subsets [12]. The former subset produces large amounts of cytokines upon stimulation, acquires cytotoxicity only after prolonged activation and is enriched in secondary lymphoid organs. On the other hand, NK cells of the latter subset readily kill susceptible targets, can rapidly secrete IFN-γ upon engagement of activating receptors and are involved in the antibody-dependent cellular cytotoxicity (ADCC) mediated by the low affinity FcγRIIIA receptor CD16 [13]. Several maturation stages can be further delineated within the cytotoxic CD56^dim^ NK cell subset based on the expression of NKG2A, Killer-cell Immunoglobulin-like receptors (KIR) [14], the intermediate stage marker CD62L [15] and the terminal differentiation marker CD57 [16].

NK cells in cHL patients at diagnosis are decreased in frequency in the TME [17] as well as in absolute numbers in the peripheral blood [10] compared to in healthy controls (HC). Furthermore, cytotoxicity toward the erythroleukemic K562 [18, 19] and the EBV- cHL L428 [20] cell lines is impaired in NK cells of cHL patients compared to NK cells of HC. Several lines of evidence suggest that NK cells found within the cHL tumor are functionally impaired [21]. Furthermore, exhausted PD-1^+^ NK cells are expanded in the peripheral blood of cHL patients at diagnosis independently of the EBV status of the tumor [22].

Thus, in the light of increasingly available adjunctive immune treatments for cHL [4] we sought to phenotypically characterize NK cells from patients with EBV+ and EBV- cHL at diagnosis and to functionally test their reactivity toward EBV-infected B cells.

## Material and methods

### Study design and human samples

Newly diagnosed and treatment naïve adult patients with cHL were prospectively enrolled at the Hematology Department of Gustave-Roussy between October 2015 and July 2018. cHL cases were classified according to the Ann Arbor staging. The cHL histological subtype was defined according to the WHO classification. The presence of EBV in the HRS cells was assessed by EBER-FISH and was not communicated until completion of the phenotypic part of the study. Serologies for HIV and EBV were performed in all cHL patients as part of the baseline evaluation. The EBV-seropositive (i.e., having serological evidence of prior infection with EBV) cHL patients of the study were either designated EBV+ (presence of EBV within the tumor; EBV-seropositive EBV+ cHL) or EBV- (absence of EBV within the tumor; EBV-seropositive EBV- cHL). The EBV-seronegative, i.e., not infected with EBV, cHL patients are EBV- cHL (EBV-seronegative and absence of EBV within the tumor). Five healthy adult donors (HC) living in the same area as the cHL patients (age range 25–49 years, 4 males, all EBV-seropositive) were enrolled secondarily in the study. The institutional ethics committee approved all protocols used and all participants provided informed consent in accordance with the Declaration of Helsinki.

### PBMC isolation

Peripheral blood samples of HL patients and HC were drawn at the Hematology Department of Gustave-Roussy, shipped to the Experimental Infectious Disease and Cancer Research laboratory in Zurich and processed for peripheral blood mononuclear cells (PBMC) and plasma isolation within 24 h of blood puncture as stated elsewhere [23].

### Quantification of EBV

Quantification of EBV DNA copy number in plasma was performed as previously described [23].

### Flow cytometry analysis

Frozen PBMCs were thawed, washed and stained with monoclonal antibodies (Supplementary Table 1) at room temperature for 15 min. LIVE/DEAD Fixable Aqua (Invitrogen) was used for dead cell exclusion. Samples were acquired on a LSR Fortessa (BD Biosciences), and all flow cytometry analyses were performed with FlowJo Version 10 software (Tree star, Inc). The NK cell subset gating strategy is depicted in the Supplementary Fig. 1.

### CD107a-based degranulation assay

The HLA class 1 negative lymphoblastoid cell line LCL721.221 (LCL221) was maintained in complete medium (RPMI-1640 medium; Sigma, Life Science) supplemented with 10% heat-inactivated FBS (Sigma), 2 mM Glutamax (Gibco by Life Technologies), 1% Penicillin Streptomycin (Gibco) and tested negative for mycoplasma. LCL221 (Cellosaurus, RRID:CVCL_6263) has been authenticated by Microsynth using STR profiling in February 2020. For the degranulation assay with rIL-2 pre-stimulation, frozen PBMCs from 7 EBV+ and 7 EBV- cHL were thawed and incubated overnight with 100 IU recombinant interleukin-2 (rIL2) per ml. For the antibody-based degranulation assay without rIL-2 pre-stimulation, frozen PBMCs from 5 EBV+ cHL, 5 EBV- cHL and 5 HC were thawed and incubated overnight without any supplementary cytokines. The next day, 1.5×10^5^ PBMC were either resuspended in complete medium (medium control) or co-cultured with LCL221 at an effector to target ratio (E:T ratio) of 10:1 for 5 h with or without the monoclonal antibody anti-CD20 rituximab (1 μg/ml, MabThera). All conditions were performed in duplicates and with addition of the monoclonal antibody anti-CD107a (LAMP-1) pacific blue (Bio Legend) at the beginning of the assay. After 1 h, monensin (1 μg/ml BD Golgi Stop; BD Pharmingen) was added to all samples. At the end of the incubation, cells were stained with mAbs and analyzed by flow cytometry. The gating strategy is depicted in the Supplementary Fig. 2. The spontaneous degranulation (medium control) from NK cell subsets was subtracted in each degranulation analysis.

### In vitro NK cell expansion

NK cells were expanded from PBMCs (4 EBV+ HL, 4 EBV- HL, 4 HC) as previously described [24–26] with some modifications. NK cells were cultured in NK cell media consisting of RPMI 1640 (Life Technologies) supplemented with 10% fetal calf serum (FCS; Biochrom), 1% penicillin/streptomycin (Thermo Fisher) and 200 IU/mL IL-2 (Preprotech). PBMCs were thawed, counted and co-cultured with irradiated (130 Gy) K562-mbIL21 feeder cells (kindly provided by Dr. Dean Lee, Nationwide Children’s Hospital, Columbus, United States) at a 1:2 NK:feeder cell ratio at day 0. On day 7 and 14, NK cells were re-stimulated with irradiated (130 Gy) K562-mbIL21 feeder cells (1:1 NK:feeder cell ratio). IL-2 (200 IU/mL) was replenished every 2–3 days. On day 20, cultured cells were CD3-depleted via immunomagnetic depletion according to manufacturer’s instructions (Miltenyi Biotech). Flow cytometric analysis of NK-cell purity and viability was performed on days 0, 14 and 20 of expansion before addition of K562mb-IL21 feeder cells.

### CD107a-based degranulation assay and ADCC assay with expanded NK cells

Degranulation assays using expanded NK cells in culture from 4 EBV+ cHL, 4 EBV- cHL and 4 HC were performed as described before but with an E:T ratio of 1:2 with 1 μg/ml rituximab. The ADCC assay was performed according to Lee-MacAry et al. [27]. Briefly, in vitro expanded and T cell depleted NK cells from 4 EBV+ cHL, 4 EBV- cHL and 4 HC were thawed and incubated overnight to rest. The next day LCL221 cells were labeled with PKH-26 (Sigma-Aldrich) and co-cultured with the expanded NK cells at a E:T ratio of 1:3, 1:1 and 3:1 for 4 h in the presence or absence of rituximab (1 μg/ml). Target cell lysis was assessed by flow cytometry on PKH-26-positive cells using TO-PRO-3 iodide (Invitrogen) staining at the end of the co-culture. The baseline target cell death was subtracted from each sample, according to the presence or absence of rituximab.

### Statistical analysis

Data were analyzed using Prism software (version 8.41.; GraphPad Software, Inc.). *P* values < 0.05 were considered significant. For the categorical data, Chi^2^ statistical test was used. For the numerical data comparing 2 groups, unpaired t test was used. For statistical analysis of 3 groups, we used the one-way ANOVA test with Tukey’s multiple comparisons test. Comparison between paired samples was assessed with the paired *t* test. Correlation studies were assessed with the Pearson test. Figures were created in Adobe Illustrator. Tables were created in Excel.

## Results

### One fourth of the cHL are associated with EBV

We prospectively enrolled 36 newly diagnosed and treatment-naïve cHL patients without known immune suppression or overt immunodeficiency. All patients were tested seronegative for HIV. The demographic and cancer characteristics of 26 EBV- cHL patients (72.2%) and 10 EBV+ cHL patients (27.7%) is shown in Supplementary Table 2, with the statistical comparison displayed in Table 1. All 10 EBV+ cHL and 25 out 26 EBV- cHL were tested seropositive for EBV antibodies. The majority (83%) of cHL was diagnosed with the nodular sclerosis subtype. None of the patients suffered from a treatment refractory cHL during the clinical follow-up. The mean age and the frequency of male patients were significantly higher in EBV+ cHL patients compared to their EBV- counterparts. Both groups did not differ in terms of frequency of histological subtypes, Ann Arbor staging, blood lymphocyte counts and EBV-seropositivity.

**Table 1:** Comparison of demographic and cancer parameters of patients with EBV-positive versus EBV-negative classical Hodgkin lymphoma. The statistical tests used are stated as footnote

	EBV-positive cHL	EBV-negative cHL	
	(*n*=10)	(*n*=26)	*P* value
**Age (years)**	48; 24; 76	35; 19; 74	0.04 ^a^
**Male sex (*n*; %)**	8; 80	10; 38.4	0.02 ^b^
**Nodular sclerosis HL (*n*; %)**	7; 70	23; 88	0.18 ^b^
**Ann Arbor stage ≥ III (*n*; %)**	5; 50	12; 46	0.83 ^b^
**Lymphocyte count (G/l)**	1.27; 0.90; 1.70	1.26; 0.40; 3.30	0.96 ^a^
**Plasma EBV DNA (copies/ml)**	3414; 0; 11,743	26; 0; 143	0.0002 ^a^
**EBV-seropositive (*n*; %)**	10; 100	25; 96	0.52

^a^unpaired *t* test (mean; min; max)

^b^chi-square test

### Reduced frequency of cytotoxic CD56^dim^ CD16^+^ NK cells in EBV+ cHL

**Fig. 1:**
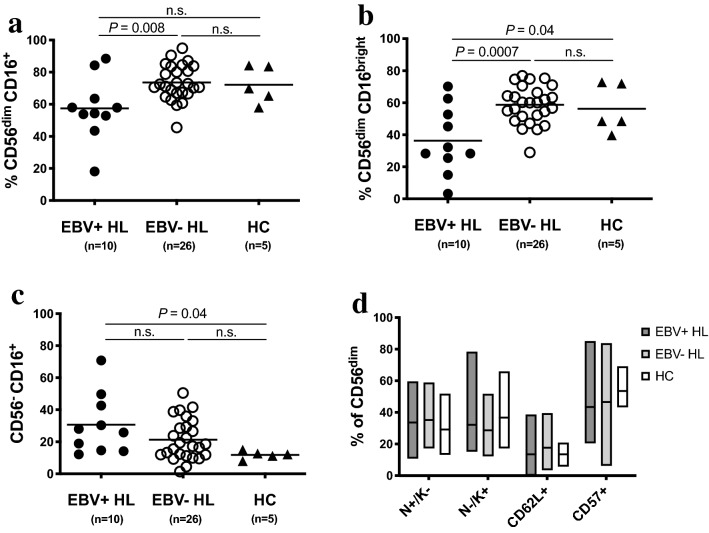
Decrease of CD56^dim^ CD16^+^ NK cell subset frequency in EBV+ HL patients compared to EBV- HL patients. Thawed PBMCs from EBV+ and EBV- HL patients and healthy controls (HC) were analyzed by flow cytometry. Frequencies of CD56^dim^ CD16^+^ **a**, CD56^dim^ CD16^bright^ **b** and CD56^-^ CD16^+^ **c** NK cells of EBV+ HL (*n*=10; filled circles), EBV- HL (*n*=26; open circles) and HC (*n*=5, triangles). **d** Frequencies of NKG2A^+^ KIR^-^ (N^+^/K^-^), NKG2A^-^ KIR^+^ (N^-^/K^+^), CD62L^+^ and CD57^+^ cells within CD56^dim^ NK cells of EBV+ HL (dark gray, *n*=10), EBV- HL (light gray, *n*=26) and HC (white, *n*=5). Horizontal lines in panels **a** to **c** indicate the mean value. Floating bars in panel **d** indicate mean and minimum and maximum values. Significance was determined by the one-way ANOVA test with Tukey’s multiple comparisons test

We assessed the frequencies of NK cell subsets in the peripheral blood of cHL patients and HC. The gating strategy is depicted in Supplementary Fig. 1. There were no differences for CD3^-^ CD56^+^ (Supplementary Fig. 3A) and CD56^bright^ CD16^-^ (Supplementary Fig. 4A) NK cells. However, we found that the CD56^dim^ CD16^+^ NK cell subset was decreased in frequency in EBV+ compared to EBV- cHL patients (Fig. 1a). CD56^dim^ CD16^+^ NK cells are composed of two functionally different subset [28], namely CD56^dim^ CD16^dim^ and CD56^dim^ CD16^bright^. EBV+ cHL exhibited a decreased frequency of CD56^dim^ CD16^bright^ NK cells compared to EBV- cHL and HC (Fig. 1b). On the other hand, we observed in EBV+ cHL an increased frequency of CD56^dim^ CD16^-^ (Supplementary Fig. 4B) and CD56^dim^ CD16^dim^ (supplementary Fig. 4C) NK cells compared to EBV- cHL. The frequency of CD56^-^ CD16^+^ was higher in EBV+ cHL compared to HC (Fig. 1c). Representative staining of three EBV+ cHL-patients, i.e., one with high frequency of CD56^bright^ CD16^-^ NK cells (left panel) and two with high frequency of CD56^dim^ CD16^-^ NK cells (middle and right panel) is depicted in supplementary Fig. 4D. The level of CD16 expression on monocytes was not reduced in EBV+ cHL patients with low frequencies of CD56^dim^ CD16^+^ NK cells (supplementary Fig. 4E). The frequencies of CD56^dim^ NK cell maturation stages defined as NKG2A^+^ KIR^-^, NKG2A^-^ KIR^+^, CD62L^+^ and CD57^+^ were comparable between the three groups (Fig. 1d). We additionally assessed the frequency of T cell subsets known to be involved in the immune response toward EBV [6]. EBV+ cHL and EBV- cHL patients exhibit similar frequencies of CD4^+^ T cells, CD8^+^ T cells, TCR δ-1^+^ (γδ﻿﻿﻿-1) T cells and TCR δ-2^+^ (γδ﻿﻿﻿-2) T cells (Supplementary Fig. 3B-E).

### Impaired CD56^dim^ NK cell-mediated rituximab-dependent degranulation in EBV+ cHL

**Fig. 2:**
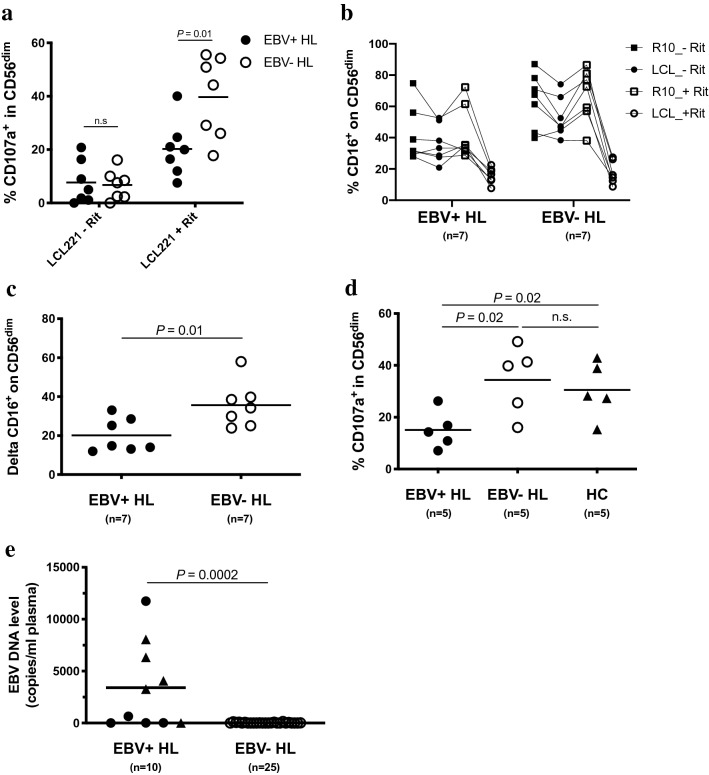
Impaired rituximab-induced degranulation in CD56^dim^ NK cells from EBV+ HL compared to EBV- HL patients. Overnight rIL-2-pre-stimulated PBMCs from 7 EBV+ HL and 7 EBV- HL patients were co-cultured with LCL721.221 cell line (LCL221) without or with 1 μg/ml of rituximab (Rit) at an effector to target ratio of 10:1 for 5 h. Frequencies of degranulating CD107a^+^ cells within CD56^dim^ NK cells **a** of EBV+(filled circles) and EBV-(open circles) HL patients. **b** Frequencies of CD16^+^ cells within CD56^dim^ of EBV+ and EBV- HL patients in different experimental conditions (medium control or LCL221; without or with 1 μg/ml rituximab). **c** CD16 downregulation between the condition LCL221 without rituximab (LCL_-Rit in Fig. 2b) and LCL221 with rituximab (LCL_+Rit) is depicted as delta. **d** PBMCs of 5 EBV+ HL, 5 EBV- HL patients and 5 healthy controls (HC) were thawed, incubated overnight without rIL2 pre-stimulation and co-cultured the next day with LCL221 in the presence of 1 μg/ml of rituximab at an effector to target ratio of 10:1 for 5 h. Frequencies of degranulating CD107a^+^ cells within the CD56^dim^ NK cells of EBV+ HL (filled circles), EBV- HL (open circles) and HC (triangles). **e** Plasma EBV DNA levels (copies/ml of plasma) of EBV+ (filled circles, *n*=10) and EBV-seropositive EBV- (open circles, *n*=25) HL patients. EBV+ HL patients were further delineated according to the clinical stage, i.e., early stage I and II (filled circles) vs. advanced stage III and IV (filled triangles). Horizontal lines indicate the mean value in Fig. 2a-e. Displayed *P* values were determined by unpaired t test for comparison of 2 groups (Fig. 2a, c and e), by the one-way ANOVA test with Tukey’s multiple comparisons test for comparison of 3 groups (Fig. 2d) and by the paired *t* test for paired samples (Fig. 2b)

We next assessed the NK cell mediated natural cytotoxicity and CD16-mediated antibody-dependent degranulation upon rIL-2 overnight stimulation and co-culture with the HLA class 1 negative LCL221. Patients from both cHL groups exhibited comparable natural cytotoxicity of the CD56^dim^ NK cell subset (Fig. 2a, left part). The addition of rituximab led in samples of EBV- cHL patients to a sixfold increase of the mean frequency of degranulating CD107a^+^ CD56^dim^ NK cells (Fig. 2a, right part), but only a 2.5-fold increase in EBV+ cHL patients. Differences in the frequencies of both effector CD56^dim^ CD16^+^ NK cells and bystander autologous CD20^+^ B cells might theoretically influence our PBMC-based antibody-dependent degranulation assay. We did confirm the decreased frequency of CD16^+^ on CD56^dim^ NK cells observed ex vivo (Fig. 1a) after overnight incubation in rIL2-containing medium of EBV+ cHL compared to EBV- cHL samples (Supplementary Fig. S5A). Moreover, we found a positive correlation between the frequency of CD16^+^ on CD56^dim^ NK cells and the antibody-dependent degranulation of CD56^dim^ upon co-culture with rituximab-coated LCL221 (Supplementary Fig. 5B). The frequency of CD56^dim^ CD16^+^ within the live PBMC is similar in EBV+ vs. EBV- cHL samples (data not shown), indicating that the effective E:T ratios applied for the 14 samples chosen for this assay are comparable between both groups. On the other hand, EBV+ and EBV- cHL samples did not differ in the frequencies of CD20^+^ CD19^+^ B cells with the live PBMC fraction (Supplementary Fig. 5C). We did not find an overall increase in the CD56^dim^ mediated degranulation in the medium control after addition of rituximab (calculated as ratio over medium control without rituximab) nor any correlation with the frequency of CD20^+^ CD19^+^ B cells (Supplementary Fig. 5D). As second read-out for antibody-dependent degranulation [29], we quantified the additional CD16 shedding of CD56^dim^ NK cells [30] upon co-culture with LCL221 cells with and without rituximab. CD16 expression on CD56^dim^ NK cells decreased upon addition of rituximab to target cells (Fig. 2b). EBV+ cHL patients exhibited a significantly reduced CD16 shedding upon co-culture with LCL221 with vs. without rituximab compared to their EBV- counterparts (Fig. 2c). We next assessed the rituximab-dependent CD56^dim^ NK cell degranulation without rIL-2 pre-stimulation of PBMC. EBV+ cHL displayed decreased frequencies of CD107a^+^ CD56^dim^ NK cells compared to EBV- cHL (Fig. 2d). Finally, we observed higher plasma EBV DNA levels in EBV+ compared to EBV- cHL patients (Fig. 2e). Patients with advanced clinical stage (Ann Arbor stage ≥ 3) were overrepresented (4 out of 5) among EBV+ cHL with high plasma EBV DNA level (>1000 copies/ml of plasma). The level of rituximab-dependent degranulation in CD56^dim^ NK cells from the 7 EBV+ cHL patients analyzed did not correlate with plasma EBV DNA levels (data not shown).

### Unaltered in vitro expansion capacity of NK cells and impaired rituximab-dependent degranulation of expanded NK cell from EBV+ cHL

**Fig. 3:**
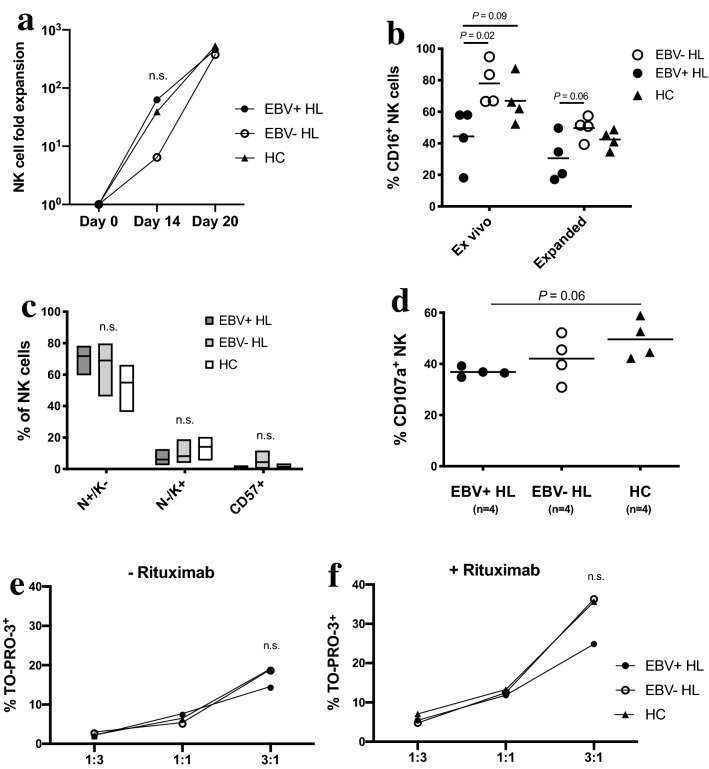
Impaired rituximab-induced degranulation in in vitro expanded NK cells from EBV+HL compared to healthy controls (HC). NK cells were expanded from PBMC of 4 EBV+HL (depicted as filled circles or dark gray floating bars), 4 EBV- HL (depicted as open circles or light gray floating bars) and 4 HC (depicted as filled triangles or white floating bars) for 3 weeks. **a** Fold expansion of CD3^-^ CD56^+^ NK cells relative to day 0 assessed by NK cell counts at day 0, 14 and 20. **b** Frequencies of CD16^+^ cells in CD3^-^ CD56^+^ were assessed at the end of expansion procedure (expanded) and compared to baseline ex vivo values (thawed PBMC) in 4 EBV+ HL, 4 EBV- HL patients and 4 HC. **c** Frequencies of NKG2A^+^ KIR^-^ (N+/K-), NKG2A^-^ KIR^+^ (N-/K+) and CD57^+^ cells within expanded NK cells in 4 EBV+ HL, 4 EBV- HL patients and 4 HC. **d** Expanded NK cells from 4 EBV+ HL, 4 EBV- HL and 4 HC were co-cultured with LCL721.221 cell line (LCL221) with 1 μg/ml of rituximab at an effector to target ratio of 1:2 for 5 h. Frequencies of degranulating CD107a^+^ cells within CD3^-^ CD56^+^ NK cells of EBV+ HL, EBV- HL and HC. (**e and f**) Thawed expanded NK cells of 4 EBV+ HL, 4 EBV- HL and 4 HC were co-cultured with PKH-26-labeled LCL221 either without **e** or with **f** 1 μg/ml of rituximab at different effector to target ratios (1:3, 1:1, 3:1) for 4 h. The frequency of dead cells among PKH-26-labeled LCL221 at the end of the co-culture was analyzed by flow cytometry using TO-PRO-3 iodide. The NK cell mediated specific killing of LCL221 was assessed by subtracting the background dead cell level in each sample. Horizontal lines indicate mean values in Fig. 3b-d. Significance between groups was determined by the one-way ANOVA test with Tukey’s multiple comparisons test in Fig. 3a-f and between paired samples by the paired *t* test in Fig. 3b

We next assessed if prolonged in vitro expansion of NK cells might rescue the reduced rituximab-dependent degranulation observed in EBV+ cHL. We expanded NK cells from 4 EBV+ cHL, 4 EBV- cHL and 4 HC for 20 days using irradiated K562 expressing IL-21 (K562-mbIL21) and rIL-2 supplementation [24]. The 3 groups did not differ in term of NK cell expansion from day 0 to day 20 (370–530-fold expansion; Fig. 3a). The mean frequency of NK cells at day 20 of expansion was comparable in each group (data not shown) and further increased between 65 and 95% after T cell depletion, except in one EBV+ cHL (15% NK cell) sample with too low cell numbers to undergo additional T cell depletion (E:T ratio in co-culture assays was maintained for this sample). The K562-mbIL21 mediated expansion led to an upregulation of CD56 (data not shown) and downregulation of CD16 expression on NK cells compared to baseline. However, only samples from EBV- cHL and HC exhibited a significant decrease in the frequency of CD16^+^ NK cells (Fig. 3b). The frequencies of NKG2A^+^KIR^-^, NKG2A^-^ KIR^+^ and CD57^+^ NK cells (Fig. 3c) did not differ between the three groups. CD62L was not detected on expanded NK cells (data not shown). We performed a degranulation assay with LCL221 coated with rituximab using a lower E:T ratio of 1:2 due to the K562-mbIL21-mediated activation of expanded NK cells. We did observe a decreased frequency of CD107a^+^ NK cells in EBV+ cHL compared to HC close to the statistical significance (mean 36% vs. 49%, one way ANOVA with Tukey’s multiple comparisons test* P*=0.06; unpaired t test *P*=0.01; Fig. 3d). We finally performed with thawed expanded NK cells a flow cytometry based-ADCC assay using PKH26 labeling of LCL221 and the To-Pro-3 iodide dead cell marker. The gating strategy is depicted in the Supplementary Fig. 6. We could not observe between the three groups any significant difference in natural cytotoxicity (Fig. 3e) nor in ADCC (Fig. 3f) with the selected E:T ratios, although a trend toward a decreased ADCC in EBV+ cHL was seen with the highest E:T ratio.

## Discussion

Here, we analyzed the phenotype and function of peripheral blood NK cells in patients with EBV+ cHL vs. EBV- cHL patients and HC. We found that EBV+ cHL patients exhibited a decreased frequency of the late-differentiated CD56^dim^ CD16^+^ subset compared to EBV- cHL patients and that this quantitative NK cell subset deficiency translated in an impaired antibody-dependent degranulation toward HLA class 1 negative LCL in EBV+ cHL patients.

The first novel finding of our study is the quantitative deficiency of peripheral blood late-differentiated cytotoxic CD56^dim^ CD16^+^ NK cells in patients with EBV+ cHL. Vari et al. [22] showed that cHL patients, of whom the EBV status of their HRS cells was not determined, have decreased frequencies and counts of circulating NK cells together with a decreased ratio of CD56^dim^ CD16^+^/CD56^bright^ CD16^-^ NK cells compared to controls. This finding taken together with our data indicate that EBV+ cHL patients might indeed exhibit a quantitative deficiency of the CD56^dim^ CD16^+^ NK cells. Furthermore, the decreased frequency of CD56^dim^ CD16^+^ NK cells cannot be explained by cryo-preservation-induced increase of CD56^dim^ CD16^-^ NK cells [31], since all peripheral blood and PBMC samples of the study were processed according to the same standard operating procedure. The deficiency was more pronounced for the CD56^dim^ CD16^bright^ NK cell subset and does not seem to be related to impaired NK cell maturation as assessed by the surface expression of NKG2A, KIR, CD62L and CD57. We previously reported that early-differentiated blood CD56^dim^ NKG2A^+^ KIR^-^ NK cells preferentially expand in patients with symptomatic acute EBV infection, i.e., infectious mononucleosis (IM) [23]. This phenotype was not observed in EBV+ cHL patients despite the presence of high levels of EBV DNA in plasma. Interestingly, CD56^dim^ NK cells from both acute IM and EBV+ cHL patients exhibited a decreased surface expression of CD16, which might be due to metalloprotease-mediated shedding triggered by cytokines or ligation of activating receptors [30]. The activation-induced downregulation of CD16 is persistent in long-term in vitro cultured NK cells [29]. Furthermore, the presence of ongoing EBV lytic replication coupled with high level of circulating IgG targeting EBV lytic antigens, as seen in both acute IM and EBV+ cHL, might lead to over-stimulation and ultimately loss of CD16 on CD56^dim^ NK cells. Finally, the quantitative deficiency of CD56^dim^ CD16^+^ NK cells in the peripheral blood of EBV+ cHL patients might be due to accumulation of this subset within the immunosuppressive TME surrounding EBV-positive HRS cells. We also observed an increased frequency of dysfunctional CD56^-^ CD16^+^ NK cells in EBV+ cHL compared to HC. Such phenotype has been described in children suffering from endemic Burkitt lymphoma with high plasma EBV DNA levels [32].

The second novel finding is that the quantitative CD56^dim^ CD16^+^ NK cell deficiency translates in an impaired antibody-dependent degranulation of CD56^dim^ NK cells from EBV+ cHL patients toward HLA class 1 negative LCL. Notably, natural cytotoxicity does not differ according to the EBV status of the cHL, implying that the activating NK cell receptors involved in the recognition of LCL and their signaling pathways are unaltered in both cHL types. The difference in age distribution in EBV+ cHL patients vs. EBV- cHL patients is an unlikely a selection bias, since ADCC function is not influenced by aging [33]. The impaired CD56^dim^ NK cell-mediated rituximab-dependent degranulation of EBV+ cHL could also be confirmed using unstimulated PBMC and was maintained when compared to HC. The frequencies of effector cells, i.e., CD56^dim^ CD16^+^ NK cells after overnight incubation remains decreased in EBV+ cHL compared to their EBV- counterparts. However, the frequencies of CD56^dim^ CD16^+^ NK cells within live PBMC are comparable in the tested samples from both groups which makes a bias of effective E:T ratio in favor of EBV- cHL very unlikely. We also did not observe any difference in the frequency of bystander CD20^+^ B cells, thus ruling out major interferences of PBMC derived B cells in this assay. Unfortunately, we were limited by the low numbers of EBV+ cHL samples and the restricted available PBMC counts in our study which prevented us to functionally test purified unstimulated NK cells. Because rIL-2-pre-stimulation of NK cell did not seem to bias the antibody-dependent degranulation between EBV+ and EBV- cHL, we took advantage of an in vitro NK cell expansion method using K562-mbIL21 and rIL-2. We could show that expanded NK cells from EBV+ cHL exhibited a decreased antibody-dependent degranulation compared to HC. The low magnitude of this decrease was possibly due to the low E:T ratio selected. Furthermore, we did not confirm an impaired ADCC using expanded NK cells from EBV+ cHL. However, the observed trend suggests that a difference might be seen at higher E:T ratios (>10:1) commonly used in many studies on NK cell-based immunotherapy. One limitation of our study is the low numbers of patients in the functional part of the experiments. We could not formally prove that CD56^dim^ CD16^+^ of EBV+ cHL patients exhibit an intrinsic deficiency in ADCC, since the activation-mediated shedding and the antibody ligation needed for cell sorting would hamper the gating and the functional testing of CD16^+^ NK cells, respectively [30]. Our study demonstrates that functional autologous NK cells from cHL patients, independently of the EBV status of the tumor, can be efficiently expanded in vitro which is a pre-requisite for the development of a NK cell based-immunotherapy targeting the HRS cells.

Elevated plasma EBV DNA levels is a feature of EBV+ cHL patients in this and other studies [34, 35] and is associated with treatment failure [36]. The origin of the circulating viral DNA is controversial and might arise from tumor-derived release of non-infectious free EBV DNA in the blood or from uncontrolled lytic replication outside of the HRS cells, which characteristically do not express EBV lytic genes. The occurrence of EBV+ cHL is preceded by unusually elevated levels of antibodies to viral capsid and early lytic antigens [37]. EBV+ cHL is associated with a specific anti-EBV antibody pattern [38]. Particularly, the combined expression of IgG specific for the two EBV lytic cycle proteins Zta and VCAp40 allows discriminating between EBV+ cHL and EBV- cHL patients. Early-differentiated CD56^dim^ NKG2A^+^ KIR^-^ NK cells can restrict EBV-infected B cells expressing lytic genes in vitro [23] and in vivo [39]. Additionally, NK cells can target EBV-infected B cells undergoing lytic replication in vitro via ADCC toward late lytic viral glycoproteins [40]. The essential role of functional CD16 signaling in the immune control of EBV lytic replication was recently demonstrated in patients with complete CD16a deficiency suffering from chronic active EBV disease [41]. Therefore, the reduced frequency of CD56^dim^ CD16^+^ NK cells and the resulting impaired antibody-dependent NK cell-mediated degranulation of EBV+ cHL described here might lead to an inefficient control of the EBV lytic replication, promoting the progression of lymphomagenesis via a yet unknown mechanism. Nevertheless, the link between the expression of lytic genes and the development of B cell cancers is supported by in vivo studies in mouse models of EBV-associated B cell lymphomas other than cHL [42–44].

Novel immunotherapies using bi-specific monoclonal antibodies targeting the CD30 antigen present on HRS cells are currently evaluated as salvage treatment for patients with refractory or relapsed cHL [4, 20]. Both strategies might rely on the presence of functional NK cells within the TME or in the peripheral blood of cHL patients. The low frequency of blood CD56^dim^ CD16^+^ NK cells found in some EBV+ cHL patients might impair the clinical efficacy to the bi-specific anti-CD30/CD16A antibody construct AFM13 currently assessed in a phase 2 study of patients with refractory or relapsed cHL (NCT02321592). Notably, AFM13-based immunotherapy in combination with NK cell-activating cytokines has been shown to expand the quantity of tumor-reactive NK cells and to improve NK cell cytotoxicity against tumor cells [45]. Thus, characterization of the EBV status of HRS cells in newly diagnosed cHL patients might be essential for future immunotherapeutic approaches that may include antibodies targeting CD16 and therefore rely on functional NK cell-mediated ADCC.

### Supplementary Information

Below is the link to the electronic supplementary material.

Supplementary file1 (PDF 5063 kb)

## Data Availability

The data that support the findings of this study are available from the corresponding author upon reasonable request.

## Abbreviations

ADCC: Antibody-dependent cellular cytotoxicity
cHL: Classical Hodgkin lymphoma
EBERs: EBV-encoded small RNAs
EBNA1: Epstein-Barr nuclear antigen 1
EBV: Epstein-Barr virus
HC: Healthy controls
HRS: Hodgkin and Reed/Sternberg
IM: Infectious mononucleosis
KIR: Killer-cell Immunoglobulin-like receptors
LCL: Lymphoblastoid cell line
LMP1/2a: Latent membrane protein 1/2a
NK cells: Natural killer cells
PBMCs: Peripheral blood mononuclear cells
rIL2: Recombinant interleukin-2
TME: Tumor microenvironment
