# Supplementary material for: Reduced frequency of cytotoxic CD56dim CD16+ NK cells leads to impaired antibody-dependent degranulation in EBV-positive classical Hodgkin lymphoma
Source: Cancer Immunol Immunother. 2021 May 15;71(1):13–24. doi: 10.1007/s00262-021-02956-x (PMC8738354; doi:10.1007/s00262-021-02956-x)
Supplement: Supplementary file 1 — Supplementary file1 (PDF 5063 kb) [file 262_2021_2956_MOESM1_ESM.pdf]

Supplementary Table 1

Antibody	Clone	Fluorophore	Company
anti-CD3	UCHT1	BV711	BioLegend
anti-CD56	B159	PE-Cy7	BD Pharmingen
anti-CD16	3G8	BV786	BD Horizon
anti-CD57	NK-1	FITC	BD Pharmingen
anti-CD57	HCD57	Pacific blue	BioLegend
anti-CD62L	DREG-56	PerCP-Cy5.5	BioLegend
anti-KIR2DL1/2DS1	EB6B	APC	Beckman Coulter
anti-KIR2DL2/2DL3/2DS2	GL183	APC	Beckman Coulter
anti-KIR3DL1	DX9	APC	BioLegend
anti-NKG2A	Z199	PE	Beckman Coulter
anti-CD107a	H4A3	pacific blue	BioLegend
anti-CD4	OKT4	PE	BD Pharmingen
anti-CD8	SK1	PE-Cy7	BioLegend
anti-TCR δ-1	TS-1	FITC	Thermo Fisher
anti-TCR δ-2	B6	PerCP	BioLegend

Supplementary Table 2

Patients	Age (years)	Sex	cHL type (histology)	cHL Stage	Plasma EBV DNA (copies/ml)	Outcome
**EBV-positive HL**						
1	37	male	NS[a]	I	21	CR[d]
2	24	female	NS	II	0	CR
3	35	female	NS	II	18	CR
4	68	male	NS	IV	0	death (pneumonia)
5	51	male	NS	IV	8057	CR
6	44	male	NS	IV	3264	lost to follow-up
7	76	male	NS	IV	6333	CR
8	52	male	MC[b]	III	4066	CR
9	29	male	MC	II	647	CR
10	65	male	Inc[c]	II	11743	death (pneumonia)
**EBV-negative HL**						
1	34	male	NS	I	0	CR
2	22	male	NS	II	0	CR
3	48	female	NS	II	0	CR
4	20	female	NS	II	0	CR
5	29	female	NS	II	0	CR
6	23	female	NS	II	0	CR
7	29	female	NS	II	0	CR
8	34	female	NS	II	56	CR
9	30	male	NS	II	82	CR
10	61	female	NS	II	0	CR
11	51	female	NS	II	125	CR
12	74	female	NS	II	16	CR
13	28	female	NS	II	0	CR
14	40	female	NS	III	17	CR
15	21	male	NS	III	0	CR
16	24	female	NS	III	143	CR
17	22	male	NS	IV	13	CR
18	48	female	NS	IV	0	CR
19	22	female	NS	IV	0	CR
20	55	male	NS	IV	71	CR
21	19	male	NS	IV	78	CR
22	33	male	NS	IV	0	CR
23	30	male	NS	IV	0	CR
24	29	female	MC	II	0	CR
25	19	male	MC	III	0	CR
26	74	female	Inc	III	57	CR

[a] NS: nodular sclerosis; [b] MC: mixed cellularity; [c] Inc: inclassable; [d] CR: complete remission

Supplementary Fig. 1

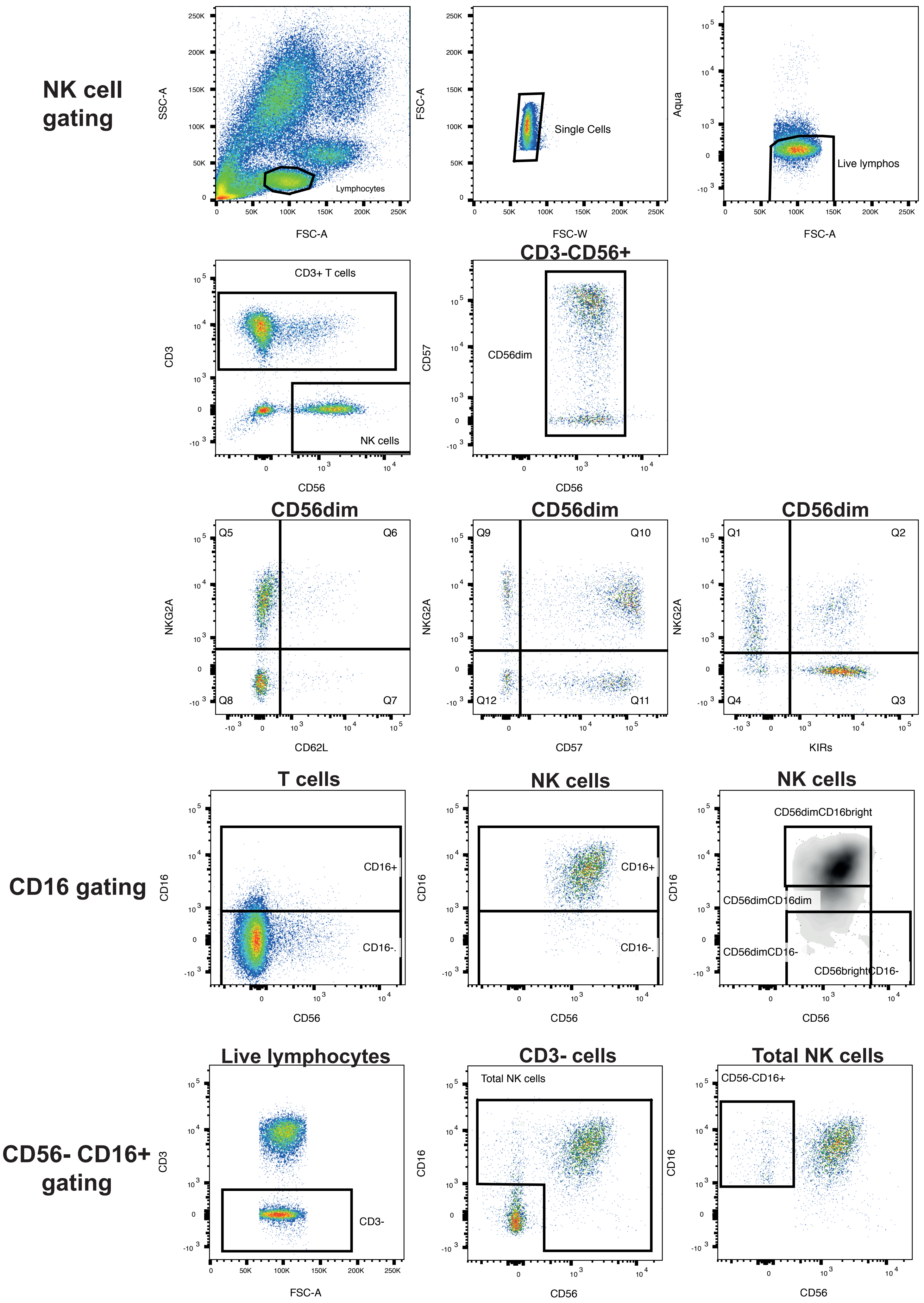

Supplementary Fig. 2

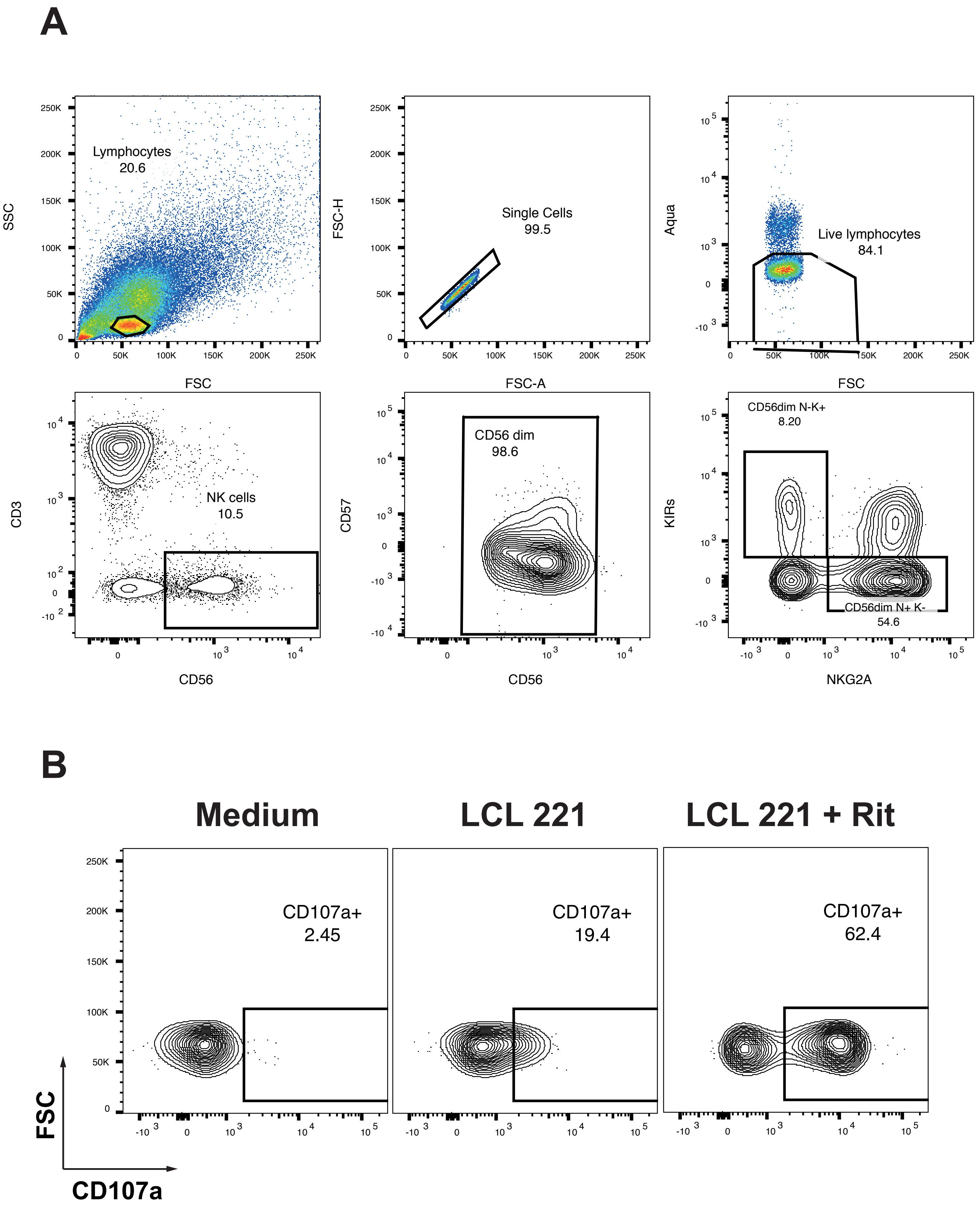

Supplementary Fig. 3

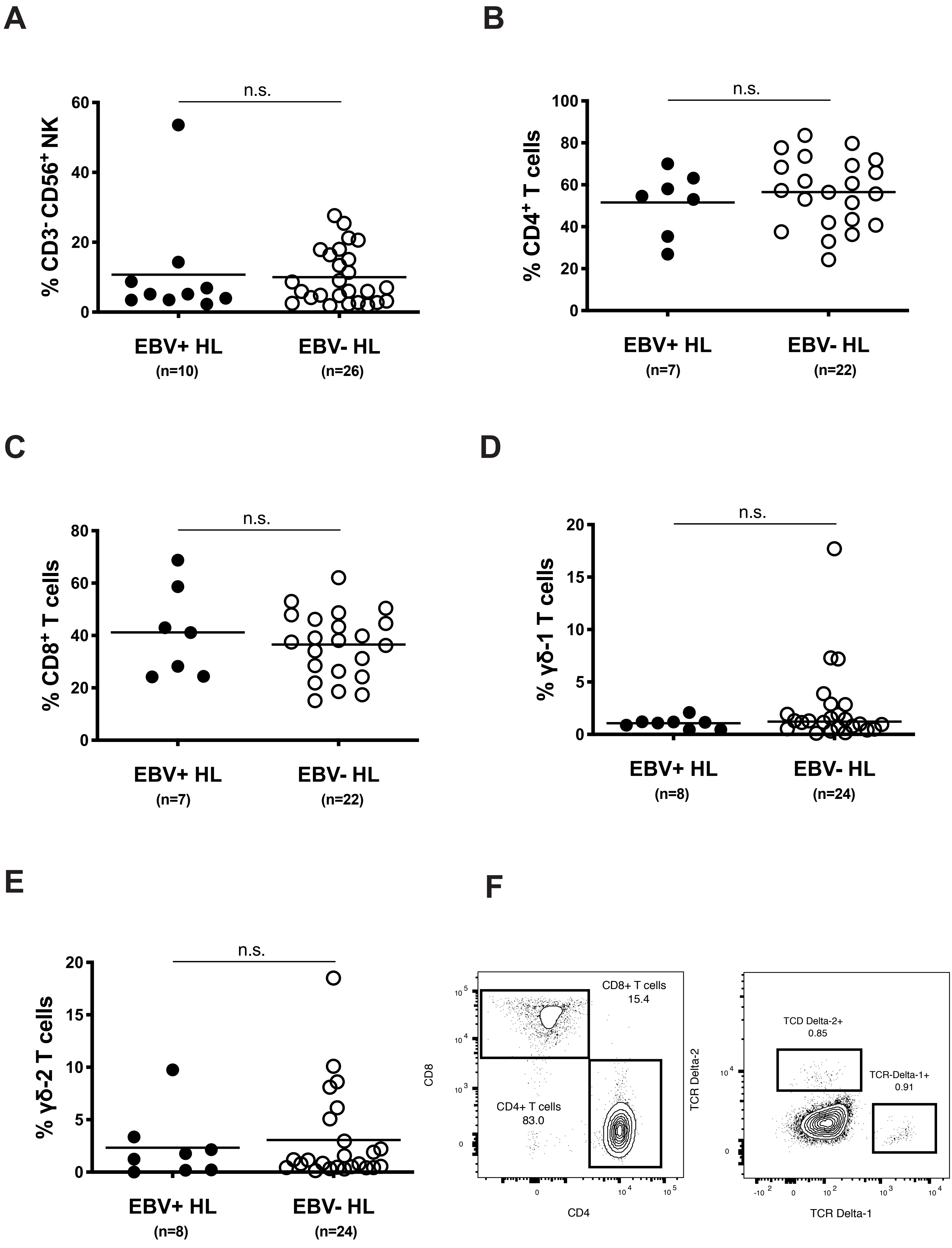

Supplementary Fig. 4

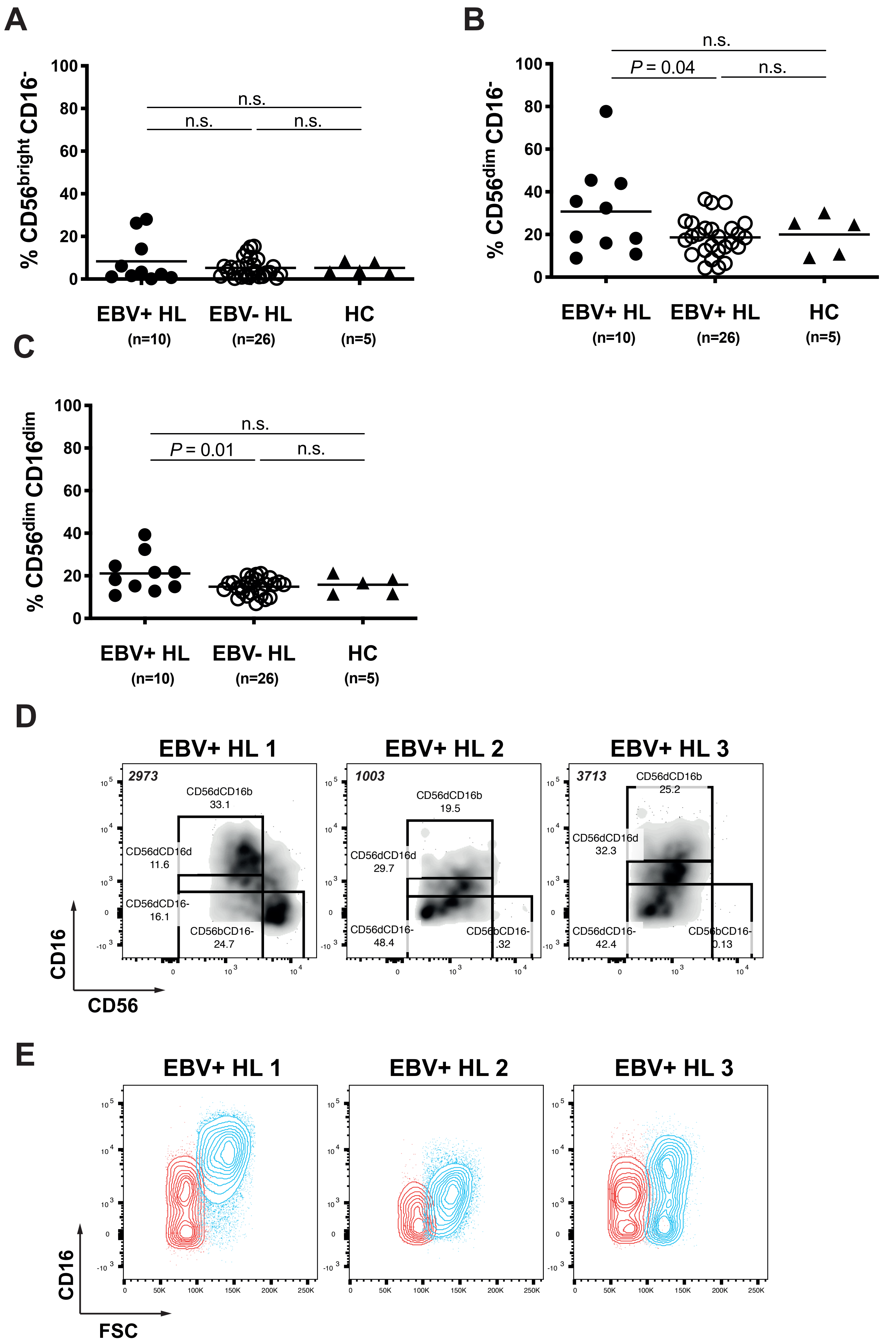

Supplementary Fig. 5

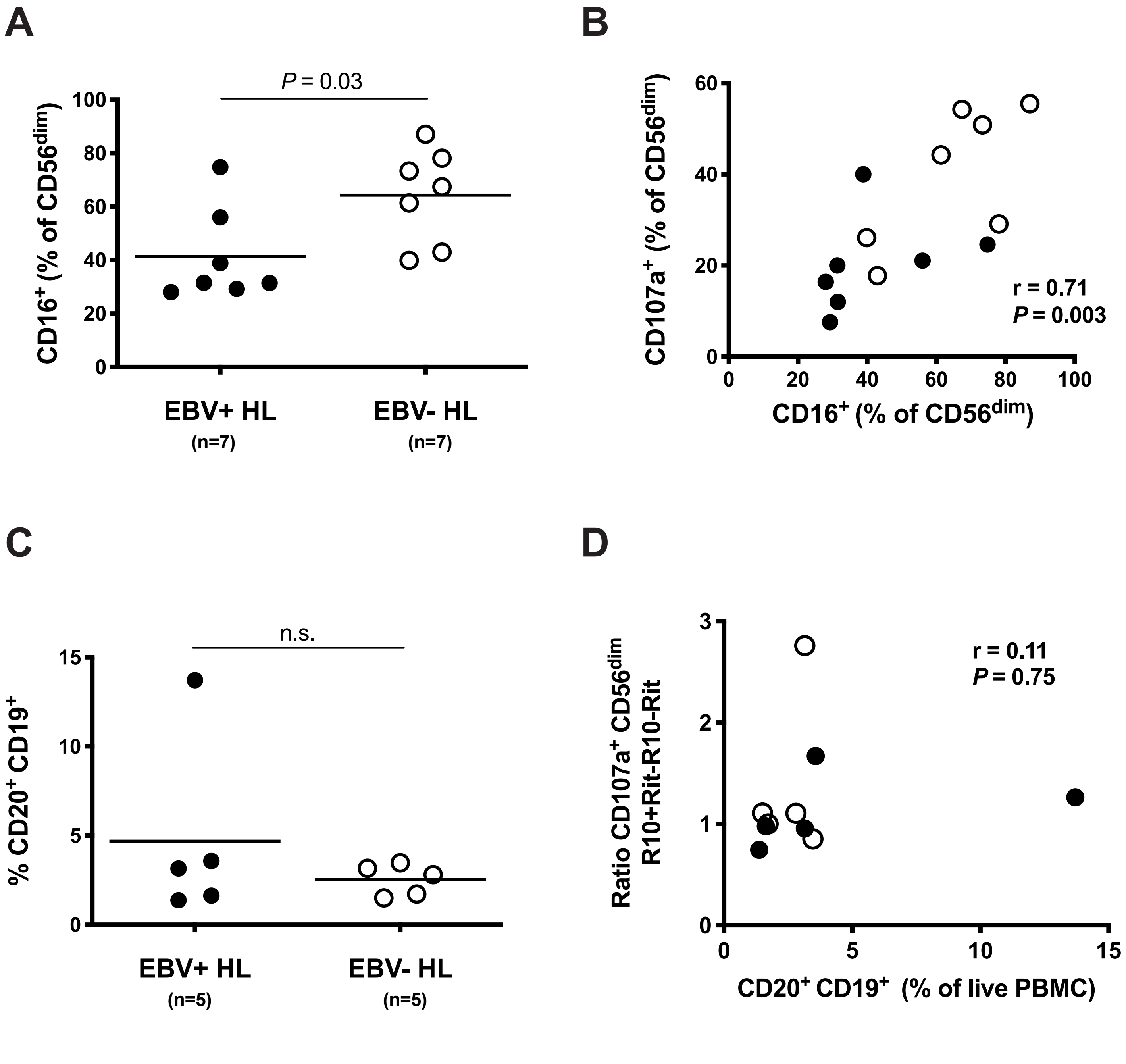

Supplementary Fig. 6

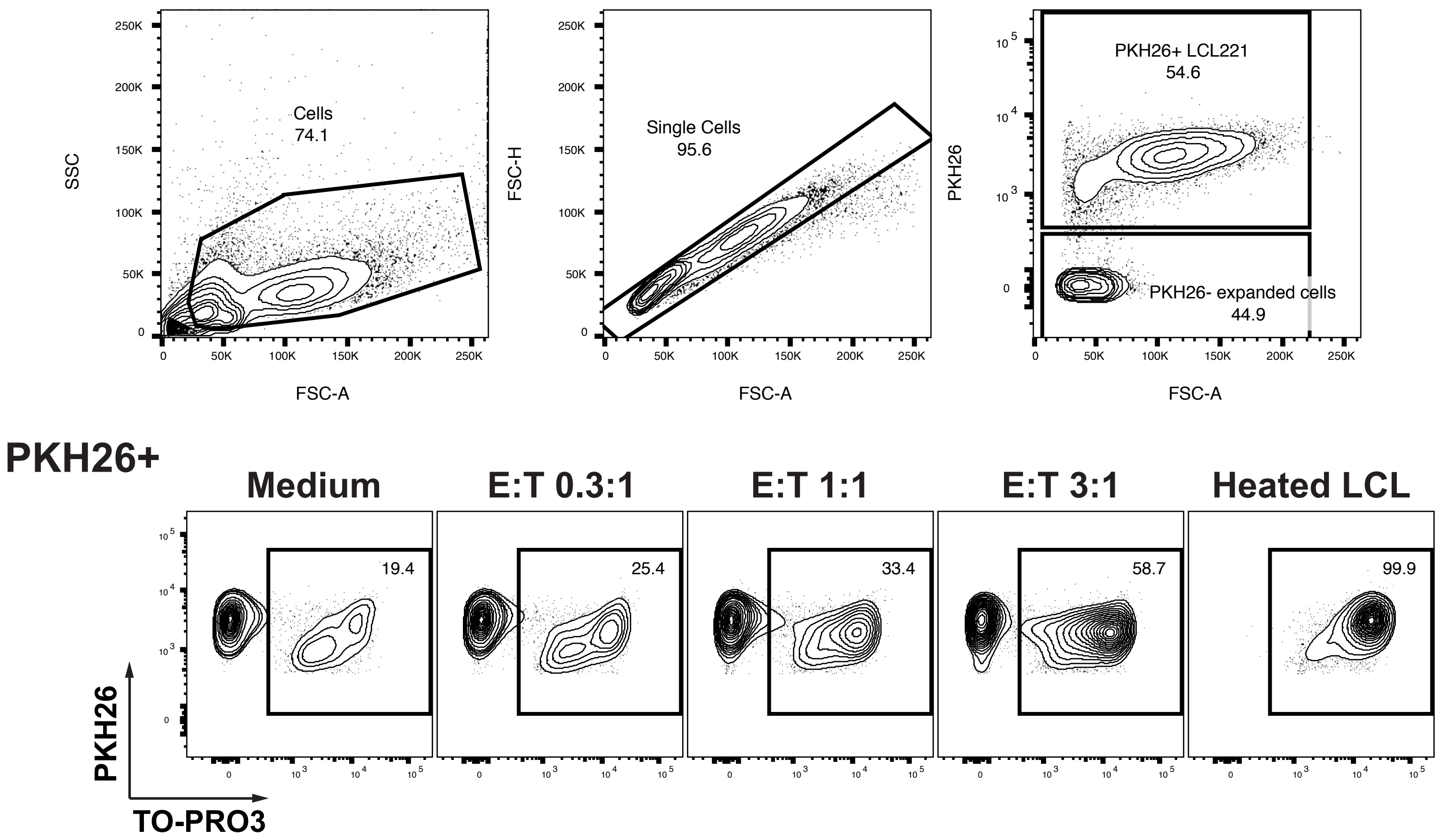

Supplementary Material – Figures legends

Supplementary Table 1 List of monoclonal antibodies used for phenotypic and functional analysis of NK cells. The clones, fluorophores and manufacturers (companies) are mentioned in the table.

Supplementary Table 2 Characteristics of patients by age, sex, cHL histological type and stage, plasma EBV DNA levels and outcome according to the EBV status (positive = presence vs. negative = absence) of the tumor biopsy. EBV-positive and EBV-negative cHL patients are depicted in the upper and lower part of the table, respectively. Abbreviations: NS, nodular sclerosis; MC, mixed cellularity; Inc, inclassable; CR, complete remission

Supplementary Fig. 1 Gating strategy of phenotypic flow cytometry analysis of $CD56^{dim}$ NK cell subset (upper part). The CD56 gate delineating $CD56^{bright}$ from $CD56^{dim}$ NK cell was assessed on the plot CD56 vs. CD57 allowing a clear separation of both NK cell subsets. The CD16 gating (intermediate part) was performed by setting the gate on $CD3^+$ T cells and transpose it to NK cell. The $CD16^{dim}$ and $CD16^{bright}$ gates were set on Flow Jo using the density plot with smoothing. The gating of $CD56^-$ $CD16^+$ NK cells (lower part) was performed separately by gating first on $CD3^-$ T cells, then on total NK cells including $CD56^+$ and $CD56^-$ $CD16^+$ cells. We acquired at least 1000 events of live NK cells at the flow cytometer.

Supplementary Fig. 2 (**A**) Gating strategy of flow cytometry-based CD107a degranulation assay. (**B**) Representative staining of CD107a on $CD56^{dim}$ NK cells. The numbers indicate the frequency of degranulating $CD107a^+$ NK cells in the medium control (left plot), co-culture with LCL721.221 (LCL 221; middle plot) and LCL 221 + rituximab (LCL 221 + Rit; right plot).

Supplementary Fig. 3 Frequencies of total $CD3^-$ $CD56^+$ NK cells and $CD3^+$ T cell subsets in EBV+ compared to EBV- HL patients. Thawed PBMCs were analyzed by flow cytometry. Frequencies of $CD3^-$ $CD56^+$ NK cells within live lymphocytes (**A**), $CD4^+$ T cells (**B**), $CD8^+$ T cells (**C**), TCR $\delta\text{-}1^+$ ($\gamma\delta$-1) T cells (**D**) and TCR $\delta\text{-}2^+$ ($\gamma\delta$-2) T cells (**E**) of EBV+ (filled circles) and EBV- (open circles) HL patients. Horizontal lines indicate mean values in supplementary Fig. 3A-E. Significance was determined by unpaired t test. (**F**) Representative staining of CD8, CD4, TCR δ-1 and TCR δ-2 on gated $CD3^+$ T cells.

Supplementary Fig. 4 Frequencies of $CD56^{bright}$ $CD16^-$ (**A**), $CD56^{dim}$ $CD16^-$ (**B**) and $CD56^{dim}$ $CD16^{dim}$ (**C**) NK cells in EBV+ HL (filled circles), EBV- HL (open circles) and healthy controls (HC; filled triangles). (**D**) Representative flow cytometry staining depicting CD56 vs. CD16 within the $CD3^-$ $CD56^+$ NK cell gate in 3 EBV+ HL (1-3) patients with low frequency of $CD56^{dim}$ $CD16^{bright}$ NK cells. The following NK cell subset gates are displayed: $CD56^{bright}CD16^-$ (CD56bCD16-), $CD56^{dim}CD16^-$ (CD56dCD16-), $CD56^{dim}CD16^{dim}$ (CD56dCD16d) and $CD56^{dim}CD16^-$ (CD56dCD16b). The exact numbers of event in the NK cell gate are reported in the upper left angle of each plot. (**E**) The CD16 staining within NK cells (red contour plot) and gated live monocytes (blue contour plot) are depicted on the same plot for the same HL patients (**E**) as in (**D**).

Supplementary Fig. 5 PBMCs of 7 EBV+ HL and 7 EBV- HL patients were thawed and incubated overnight in rIL-2-containing medium to assess the natural cytotoxicity and the rituximab-dependent degranulation toward LCL721.221. PBMC samples in the medium control without addition of rituximab (negative control) were analyzed by flow cytometry to quantify the frequencies of $CD16^+$ cells on $CD56^{dim}$ NK cells (**A**) and $CD20^+$ $CD19^+$ B cells

(**C**) within the live PBMCs from 7 patients, respectively 5 patients in each group. Both frequencies were compared between EBV+ and EBV- HL patients. (**B**) Correlation of the frequencies of CD16^{+} cells on CD56^{dim} NK cells with frequencies of CD107a^{+} CD56^{dim} NK cell mediated rituximab-dependent degranulation of pooled data from EBV+ and EBV- HL patients. (**D**) Correlation of the frequencies of CD20^{+} CD19^{+} B cells within live PBMCs with CD107a^{+} CD56^{dim} NK cell ratio in the medium control with rituximab over the medium control without rituximab. This correlation was assessed using pooled data from EBV+ and EBV- cHL patients. The EBV+ cHL (filled circles) and the EBV- cHL (open circles) can be differentiated on correlation plots. Horizontal lines indicate mean values in **A** and **C**. Significance was determined by unpaired t test in **A** and **C**. The r coefficient and significance were assessed with the Pearson correlation test in **B** and **D.**

Supplementary Fig. 6 Gating strategy of flow cytometry based ADCC assay using PKH26 target cell labeling and dead cell staining with To-Pro-3 iodide. K562-mbIL21 expanded NK cells were co-cultured with LCL721.221 (LCL221) for 4 hours. To-Pro-3 was added at the end of the co-culture and the samples were analyzed by flow cytometry. For the gating strategy, the debris were excluded on the FSC/SSC plot, followed by gating on singlets and then on PKH-26^{+} LCL221. The frequencies of To-Pro-3^{+} cells within the PKH-26^{+} LCL221 according to different conditions (medium control, effector:target ratio of 1:3, 1:1 and 3:1, heated LCL221 as positive control for cell death) are depicted in the lower part.
